# FluoroTome 1: An Apparatus for Tomographic Imaging of Radio-Fluorogenic (RFG) Gels

**DOI:** 10.3390/polym11111729

**Published:** 2019-10-23

**Authors:** John M. Warman, Matthijs P. de Haas, Leonard H. Luthjens, Tiantian Yao, Julia Navarro-Campos, Sölen Yuksel, Jan Aarts, Simon Thiele, Jacco Houter, Wilco in het Zandt

**Affiliations:** 1 Department of Radiation Science and Technology, Faculty of Applied Sciences, Delft University of Technology, Mekelweg 15, 2629 JB Delft, The Netherlands; m.p.dehaas@tudelft.nl (M.P.d.H.); l.h.luthjens@tudelft.nl (L.H.L.); tiant.yao@gmail.com (T.Y.); julianavarro.c@gmail.com (J.N.-C.); S.Yuksel@tudelft.nl (S.Y.); 2 4PICO B.V., Jan Tinbergenstraat 4B, 5491 DC Sint-Oedenrode, The Netherlands; Jan@4pico.nl (J.A.); Simon@4pico.nl (S.T.); Jacco@4pico.nl (J.H.); Wilco@4pico.nl (W.i.h.Z.)

**Keywords:** fluorescent gels, radio-fluorogenic (RFG) gel, tomographic fluorescence imaging, polymer-gel radiation dosimetry, 3D radiation dosimetry

## Abstract

Radio-fluorogenic (RFG) gels become permanently fluorescent when exposed to high-energy radiation with the intensity of the emission proportional to the local dose of radiation absorbed. An apparatus is described, FluoroTome 1, that is capable of taking a series of tomographic images (thin slices) of the fluorescence of such an irradiated RFG gel on-site and within minutes of radiation exposure. These images can then be compiled to construct a 3D movie of the dose distribution within the gel. The historical development via a laboratory-bench prototype to a readily transportable, user-friendly apparatus is described. Instrumental details and performance tests are presented.

## 1. Introduction

Several bulk media have been proposed for the creation of three-dimensional images of the energy deposited in materials by complex fields of high-energy photon or particle radiation [1]. The need for such media has been driven by the increasing sophistication and subtlety of dose delivery in radiotherapy clinics and the requirement for quality control of treatment protocols and equipment [2,3,4]. The use of such media as phantoms for the training of clinical personnel is an additional, important role they may play.

The media proposed are based on physico-chemical effects induced at a molecular level that change the local optical, dielectric or nuclear spin properties of the medium. The changes are spatially “fixed” in a quasi-rigid-gel or polymer matrix. The apparatus and data handling required to derive 3D dose mapping using such systems has been covered in the literature [1,5]. None of the methods reviewed are capable of rapid, on-site data analysis and no single method has received universal acceptance in the clinic. Multiple-arrays of solid-state devices or multi-layers of radiochromic films have also been proposed but are incapable of providing the sub-millimeter spatial resolution desired.

Here we describe a readily transportable, user-friendly apparatus that has been developed to allow on-site analysis of radio-fluorogenic (RFG) gel media within minutes of radiation exposure. The use of RFG gels to monitor dose deposition by a variety of radiation sources, from a brachytherapy radioisotope to an 80 MeV proton beam, has been recently reviewed [6]. In these early measurements, images of the *bulk* medium were made. An advance has been the incorporation of slits, forming a thin sheet of UV excitation light, that allowed the apparatus to make tomographic (multiple slice) images of the radiation-induced fluorescence within the gel. These slices can then be compiled to form 3D images that can be displayed in video mode [7,8]. This report describes the design, construction and testing of an apparatus based on this laboratory prototype by a firm that specializes in devices based on UV solid state sources.

## 2. Historical Background

The first application of a bulk radio-fluorogenic gel to monitor a non-homogeneous radiation field was reported in 2011 by Warman et al. [9]. Application to a variety of radiation sources, including x-ray beams, proton beams and radio-isotopic seeds followed [6]. These measurements of bulk fluorescence were carried out initially with an extremely simple set-up consisting of two Mercury discharge lamps and a standard Ricoh “Caplio RX” digital camera, as illustrated in Figure 14 of reference 6. The mercury lamps were eventually replaced by LED lamps from 4PICO.BV. These had a 16 cm, linear array of UV light emitting diodes with a linear collimating Fresnel lens. Using these lamps pseudo-3D images of bulk 40 × 40 × 40 mm^3^ gel samples irradiated with multiple proton beams were made, as shown in Figure 2 of reference [10].

**Figure 1:**
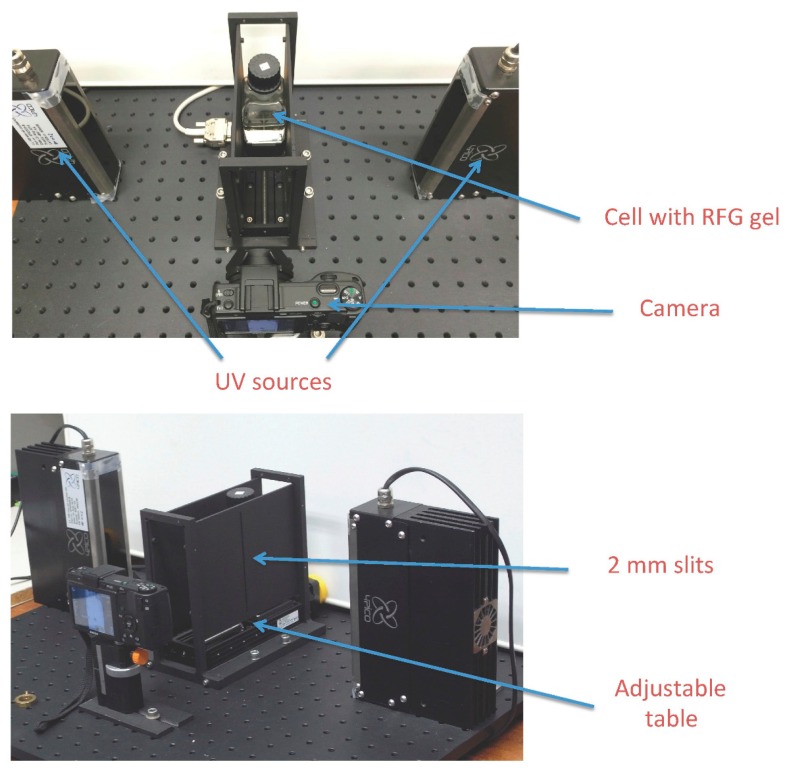
The bench-top prototype to FluoroTome 1.

The development to the prototype of the present apparatus required the introduction of plates on both sides of the sample that had slits which restricted the UV light to a thin sheet passing through the sample. A translatable stage was incorporated to transport the sample through the UV sheet and multiple images were taken of the fluorescence as a function of position. This procedure has been fully described and illustrated in reference 7. A photograph of the bench-top prototype is shown in Figure 1.

**Figure 2:**
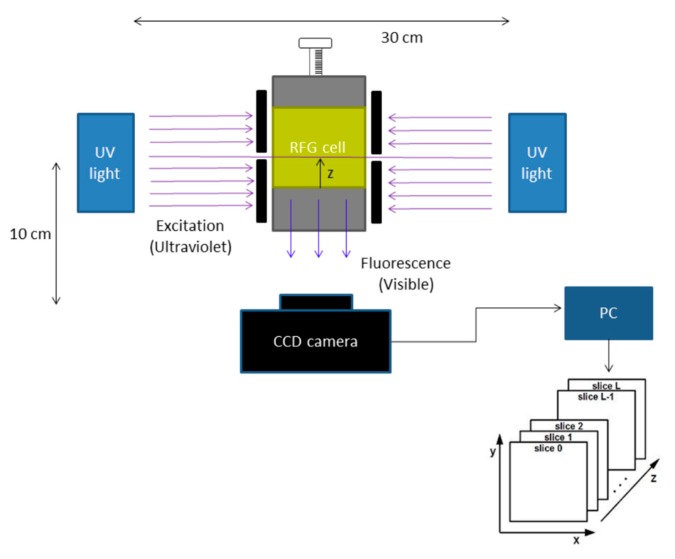
A schematic of the prototype shown in Figure 1 illustrating the mode of operation.

The operation of the prototype in making tomographic, “sliced” images of the fluorescence of a gel sample is illustrated schematically in Figure 2. The application to crossed-beam irradiation of an RFG gel [7] and to irradiation of an eye phantom with a 3 mm X-ray beam [8] have been reported.

What is hidden in Figure 1 is the spaghetti-like complexity of power supplies and cable connections that made transportation to and operation at distant venues difficult. The prototype also required a dark room in the vicinity of the measurements for fast read-out. Clearly a compacter, transportable and user-friendly apparatus was required.

## 3. The FluoroTome 1 Apparatus

**Figure 3:**
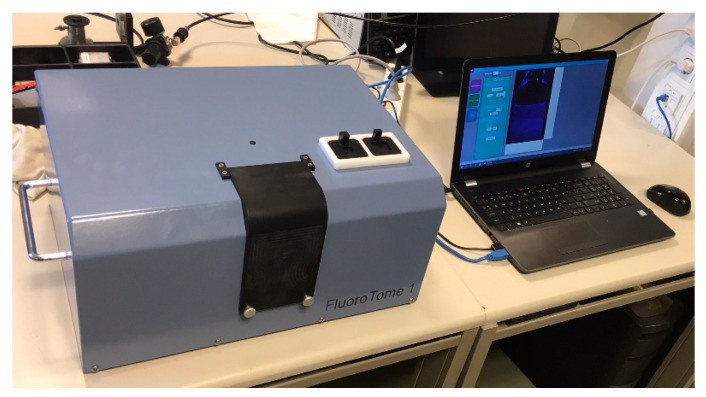
The complete FluoroTome 1 apparatus including laptop with software for camera and stage control and data management.

FluoroTome 1, shown in Figure 3, was designed and constructed by 4PICO BV (Sint Oedenrode, The Netherlands) in consultation with members of the Fluorodose project at the Reactor Institute of the Technical University of Delft, which provided the funding.

The apparatus was delivered on September 10th 2018 and further development of the software continued into January 2019.

**Figure 4:**
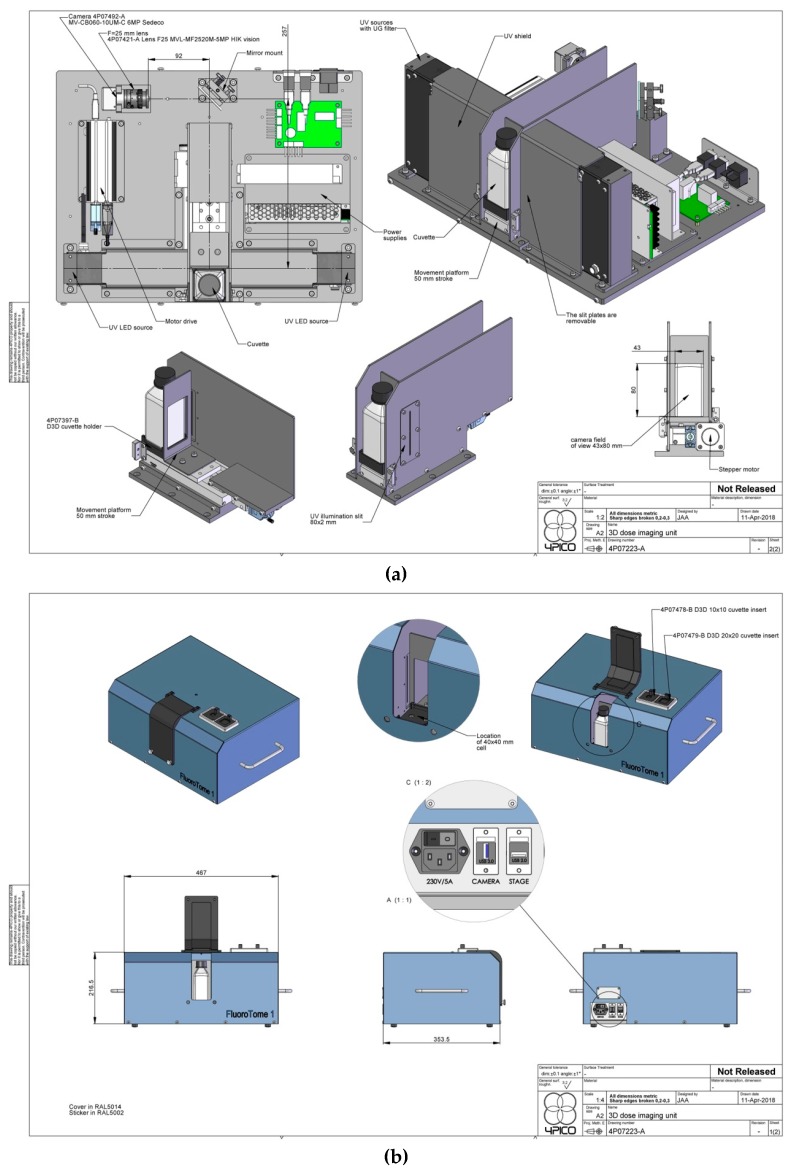
(**a**) (**upper**) and (**b**) (**lower**) are schematic drawings of the internal and external details of the apparatus respectively. See supplemental information for downloadable .jpg files.

**Figure 5:**
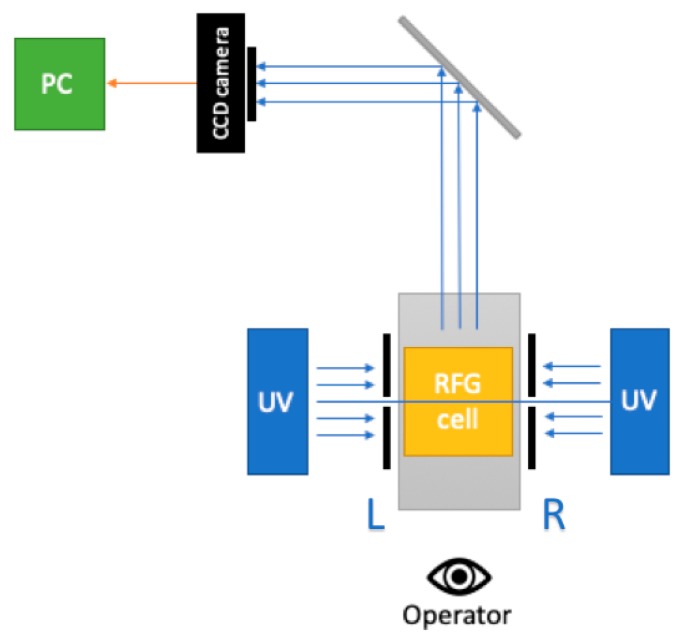
A schematic of the basic design of FluoroTome 1 with LED-array UV lamps, a CCD camera, and a translation stage in grey.

Detailed schematics of the external and internal aspects of the apparatus are shown in Figure 4a,b with downloadable versions given in the supplemental information. A schematic of the basic layout is shown in Figure 5. This is somewhat different to that of the prototype shown in Figure 2, with the major difference being the placement of the camera on the opposite side of the sample to the observer with a 45-degree mirror acting to turn the fluorescent image of the sample through 90 degrees. This results in compactness and allows for a longer, 300 mm, image distance and a greater depth of field. As can be seen in the figure, because of the mirror, the left side of an image as taken by the camera is also the left side as viewed by the operator from the front.

The basic functioning of the apparatus is as follows: with the light-tight flap open a 40 mm square borosilicate glass cell containing a fluorescent gel or solution is positioned in a holder on the translation stage. For the smaller (20 or 10 mm) sized cells the adapters shown adjacent to the flap in Figure 3 are used. The flap is closed and the translation stage with cell is moved to a given position with respect to the UV slits using the laptop software. The maximum travel of the stage is 50 mm; ±25 mm about the central position. An image of the fluorescence is taken and the raw file downloaded via a USB3 cable to the laptop where it is converted to 16-bit TIFF or 8-bit JPEG files. The camera exposure time can be varied by the user but the camera gain and color balance are fixed. Multiple images can be made in scanning mode with the width of the scan about the central position and the total number of images chosen. The images can then be individually analyzed using ImageJ or other software applications for compiling and 3D reconstruction of multiple tomographic images. A user manual has been compiled and is included in the supplemental information.

**Figure 6:**
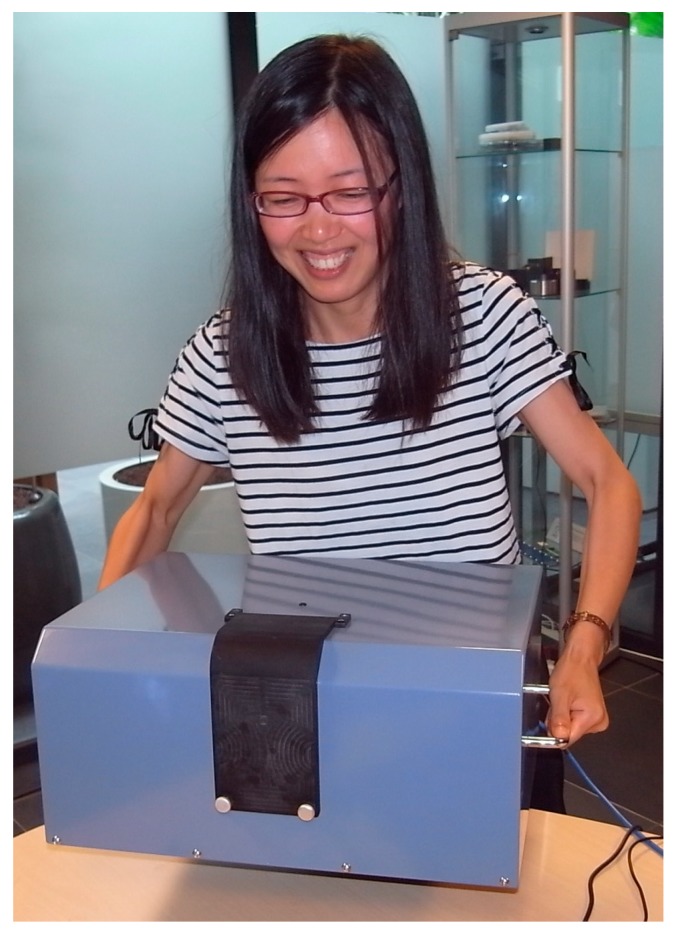
Portability test of FluoroTome 1.

The main apparatus can be lifted and carried by a moderately fit person, see Figure 6. For transport over substantial distances a more stable and reliable form of carriage is advised in view of the somewhat critical optical alignments involved. A small bench space with two 220 V outlets is sufficient for operation. Darkroom facilities are not necessary. This makes it possible to carry out on-site measurements and analysis of the radiation-induced fluorescence in RFG gels within minutes of their radiation exposure.

## 4. Test Procedures

Prior and subsequent to delivery tests have been carried out on the various components, the operational functions and the data acquisition and handling operations of the complete apparatus. These tests are described in this section. Use of the apparatus for preliminary measurements on an irradiated gel sample are the subject of Section 5.

### 4.1. UV Excitation

The collimated, linear-array UV-LED lamps used were designed and constructed by 4PICO. They consist of a 16 cm long, vertical linear-array of 22 LEDs, covered by a rectangular Fresnel lens creating a 20 mm wide collimated beam. The individual LEDs were provided by High Power Lighting Corporation, New Taipei City, Taiwan (model HPL-H44DV1C0, High efficiency 3W UV LED). Passage through the slits results in a uniform sheet of UV light 2 mm thick, 60 mm high and 60 mm wide in the cell containment region. The output spectrum of the UV lamps is Gaussian with a wavelength maximum at 384 nm and a half-width (FWHM) of 10 nm. A 1 mm thick UG-1 polished optical filter (Phillips Safety Products, Middlesex, NJ, USA) attenuated any visible-light components.

The intensity of the beam transmitted by the slits was measured as a function of height above the stage using a photodiode detector and the values are listed in Table 1. The left and right side slit intensities differ by less than 2% and the sum is constant within ±2% over the distance from 30 to 90 mm. At 20 mm, the smallest height possible using the photodiode, the intensity is 10% lower than this average. This indicated that raising the base level of the cell mounts by 20 mm would result in better uniformity of illumination, as has actually been found.

A further test of beam uniformity was carried out using a very dilute (micromolar) solution in cyclohexane of the fluorescence standard diphenyl-anthracene (DPA). This has a fluorescence maximum at approximately 400 nm (close to that of the fluorescent version of the RFG gel) with a high extinction coefficient of 14,000 L/mol.cm and a quantum yield of fluorescence close to unity. The fluorescence of such a solution, by nature of its low-viscosity, is directly proportional to the intensity of the incident excitation light. It gives therefore a measure of any spatial variation in the incident UV light intensity.

**Figure 7:**
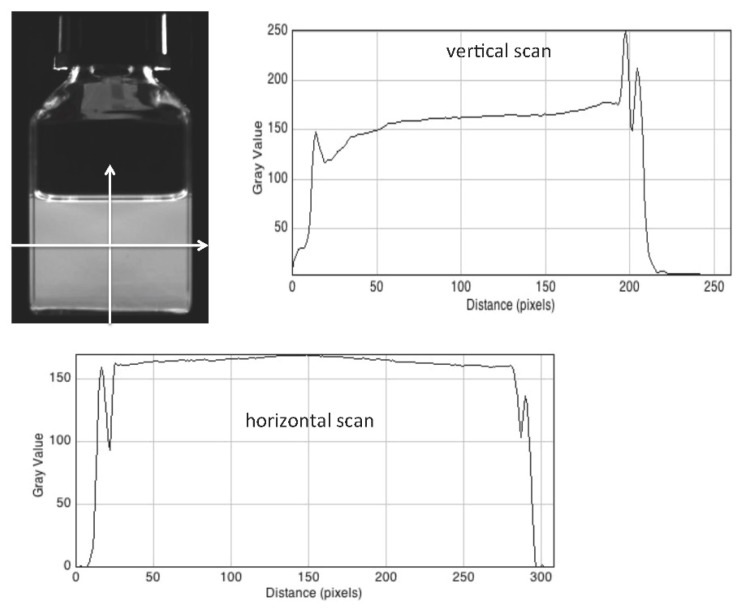
A fluorescence image of a 40 mm square cell containing 30 mm of a dilute liquid solution of diphenyl anthracene with vertical and horizontal intensity profiles made using ImageJ at 6.6 pixels per mm.

A fluorescence image of a 40 mm ID square cell containing 30 mm of a DPA solution is shown in Figure 7 together with vertical and horizontal scans of the blue pixel intensity using ImageJ. Aside from optical artifacts at the solution/air meniscus, and solution/glass interface in the former and the solution/glass/air interfaces in the latter, the intensities are seen to be fairly uniform. The horizontal intensities display an increase in the center of the cell by 5%. Since, if anything, a slight decrease would have been expected due to UV absorption, this indicates a non-uniformity on the detection side of the apparatus which should be corrected for. The levels at the left and right side of the scan are equal within 1%, in agreement with the close similarities of the photodiode measurements of the beam intensities at the left and right hand side slits given in Table 1.

**Figure 8:**
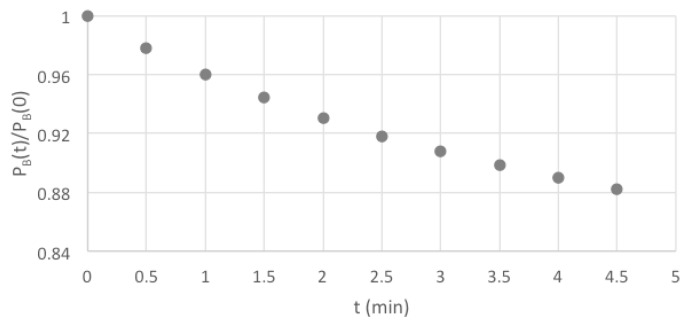
The decrease in fluorescence intensity of a DPA solution with time after turning on the UV lamps continuously.

The high-power LEDs used displayed an appreciable decrease in intensity with time after turning on due to heating. Using the photodiode detector this was found to be 13 % after 5 min. Due to this, the maximum “on time” of the lamps was automatically limited to 5 min. A test, using the fluorescent solution mentioned in the previous paragraph, was also carried out and is shown in Figure 8. The decrease in the fluorescence intensity (the blue-pixel grey-scale value, P_B_) of 13% over 5 min matched that found for the photodiode output measurements. Due to these observations the lamps were held in a default, “standby” *off* mode when carrying out measurements.

**Figure 9:**
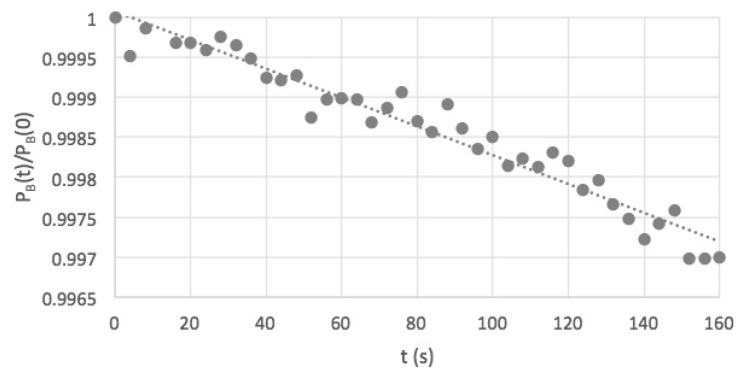
The variation in intensity of images of a DPA solution during a dummy 40 image scan without appreciable movement of the stage using the default standby mode of operation with pulsed UV illumination.

When taking an image the lamps were triggered to be *on* for only a short period (seconds) before and after the camera exposure that was set to a maximum value of 2 s. A dummy test has been carried out using the DPA solution in which a scan involving taking 40 images was carried out without the stage moving an appreciable distance. The change in the intensity of the image with scan time is shown in Figure 9 to be less than 1% over the total scan. Figure 9 also shows the statistical error from sample to sample in the scan to be extremely small.

### 4.2. Fluorescence Detection

Images were made of the cell, containing a fluorescent solution or gel, using an HIKVISION (Hangzhou, China) model MV-CB060-10UC camera with a 4P07421-A lens. An example of such an image of a fluorescent DPA solution is shown in Figure 7. When the stage is at its median position, the center of a cell is 300 mm from the camera. As can be seen in Figure 4b and Figure 5, the image is taken in reflection via a 45 degree mirror which results in the view of the image by the camera being the same as that of the operator; i.e., the left side of the camera image (viewed from the rear of the cell) is also the left side as viewed from the front by the operator.

The raw images taken by the camera are transferred directly via a USB3 cable to the laptop where they are converted to 16-bit TIFF and 8-bit “JPEG” files. These files can be uploaded to the freely down-loadable software program ImageJ [11] which is used for data analysis including RGB color separation, determination of the average pixel grey levels for a specific rectangular area of an image (as in Figure 8 and Figure 9) and making profile scans of pixel intensity (as in Figure 7).

**Figure 10:**
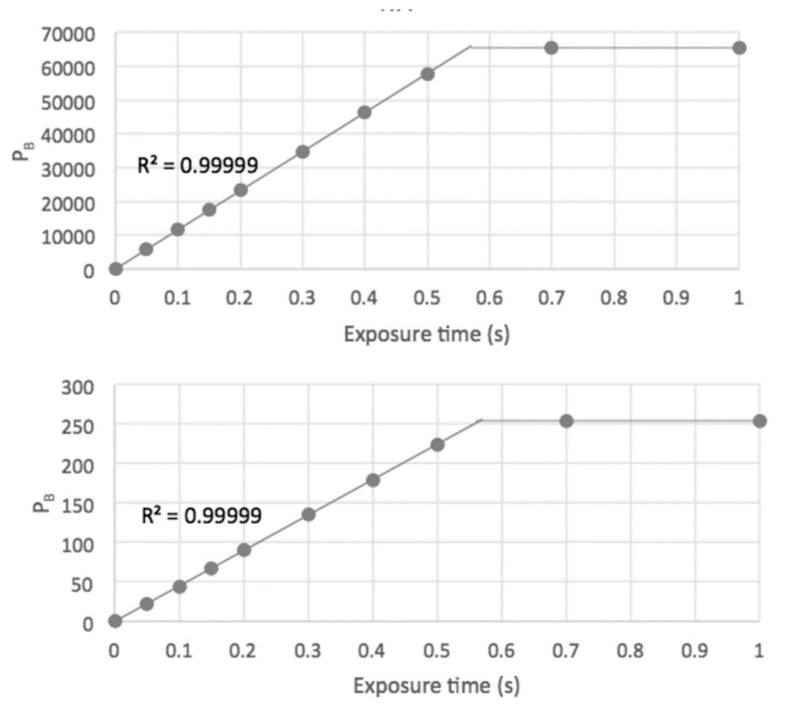
Blue-pixel grey-scale values of the 16-bit TIFF (upper) and 8-bit “JPEG” (lower) image files of an area of a fluorescent DPA solution as a function of the camera exposure time setting.

In Figure 10 blue-pixel grey-scale values are plotted as a function of the camera exposure time for a fixed area of a fluorescent DPA solution. As can be seen, both the TIFF and “JPEG” files display a very good linear dependence on the integrated photon intensity up to saturation. In fact, the “JPEG” values are exactly 8 bits down on the TIFF values indicating that the more complex JPEG corrections, usual in commercial digital cameras, have not been applied. For most purposes therefore using the 8-bit JPEG files within the linear region is sufficient and has the advantage of being considerably smaller than the 16-bit TIFF version. This is a particularly important factor when making 3D reconstructions involving many files.

**Figure 11:**
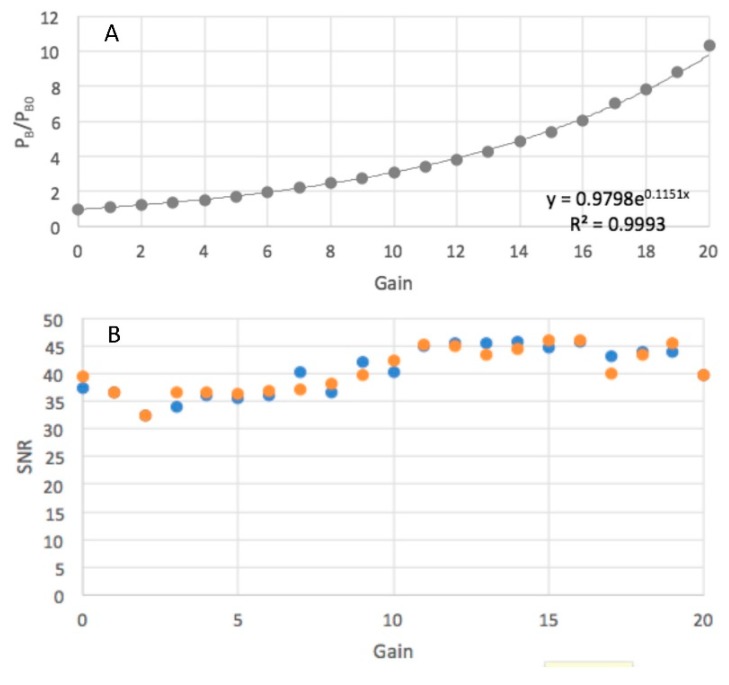
The dependence on the gain parameter of (**A)**, the pixel level and (**B)**, the signal-to-noise ratio with 16-bit images in blue and 8-bit images in orange.

The camera outputs RGB files, and the color balance can be adjusted by varying the red, green and blue pixel levels individually between 0 and 4095, i.e., 12 bits. Since the fluorescence of present interest lies in the blue spectral region (±400 nm maximum), the sensitivities were set at red = 0, green = 0, and blue = 4095. This allows files in which the blue pixels in an RGB file may be saturated to be discarded. The overall gain factor could also be varied, between 1 and 20. The effect of varying the gain on the magnitude of the output for a constant light intensity is shown in Figure 11A. Changing the gain factor did not appear to influence the signal to noise ratio as shown in Figure 11B. It is important that any changes in the gain factor or the color balance are made only by acknowledged technicians and noted. These parameter changes should not be possible in the normal user interface.

**Figure 12:**
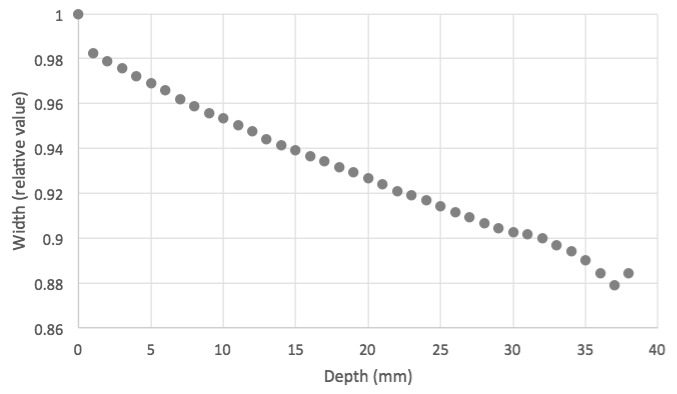
The apparent width of the cell as a function of the length of liquid dielectric between the UV sheet and the camera.

A phenomenon that must be taken into account in the scanning measurements is that the length of the dielectric medium in the cell between the fluorescent sheet and the camera changes. This has the effect of changing the apparent dimensions of the object. This can be seen in Figure 12, where the apparent width of the cell measured in pixels is plotted against the length of the dielectric layer between the fluorescent layer and the camera as the scan proceeds. The width appears to decrease by approximately 10% over the 40 mm scan. This effect should be taken into account when compiling images for 3D representation.

**Figure 13:**
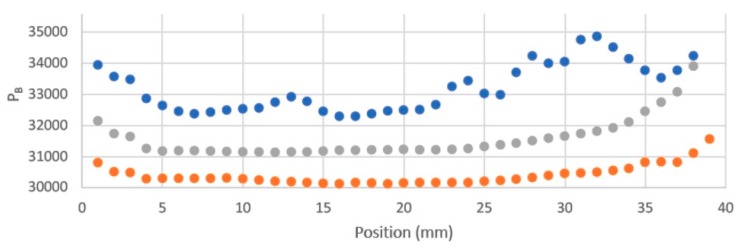
The average blue pixel level in a small area of the DPA solution as a function of the stage position. Upper, blue points: cell cap and slits uncovered; central, grey points: cell cap and shoulder masked; lower, orange points: top of slits masked.

**Figure 14:**
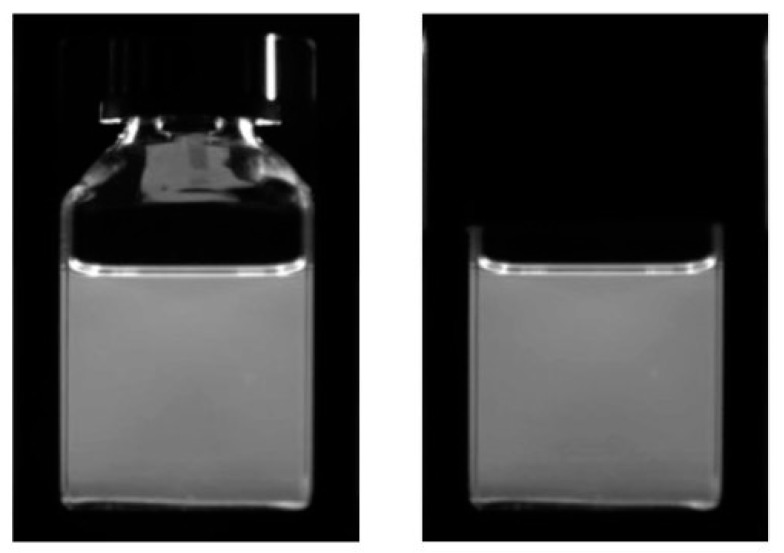
Images of the cell containing the fluorescent DPA solution. Left; no cover. Right; black paper cover over cap and shoulder of the cell.

The magnitude of the average blue pixel level in a small, defined area of the bulk of the DPA solution as a function of the scan position is shown in Figure 13. Initial results indicated a non-uniform variation in the intensity (Figure 13 blue points). Masking of either the upper part of the slits or the cap and shoulder of the cell reduced the non-uniformity and the average pixel level by approximately 5%. The additional intensity is attributed to spurious reflections from the upper, empty regions of the cell as can be seen in Figure 14 (left). A black paper, non-fluorescent mask that covers the cap and shoulder of the cell is therefore advised. Using this the intensity is found to be constant within 3% over 35 mm for a 40 mm square cell.

### 4.3. The Translation Stage

The translation stage used was a model 8MT175-50 from Standa Ltd. (Vilnius, Lithuania) with a maximum travel range of 50 mm and micrometer resolution. The scanning parameters were software controlled as described in the user manual (downloadable in the supplementary information). The position of the stage was defined with respect to the UV slits with position 25 corresponding to the center of the stage and the mounted cell. In positions 1 or 50 the stage is farthest from or nearest to the camera respectively. For scanning, a choice is made of the width of the scan about the central position, ΔW. As an example, a choice of 30 mm results in a scan from position 10 (25 − ΔW/2) to position 40 (25 + ΔW/2). The number of images to be taken during the scan, N, is then chosen with a choice of 31 resulting in images being taken every mm. A choice of 7 would result in images being taken every 5 mm. The step size between images is in general ΔW/(N − 1).

## 5. Preliminary, Irradiated RFG Gel Measurements

**Figure 15:**
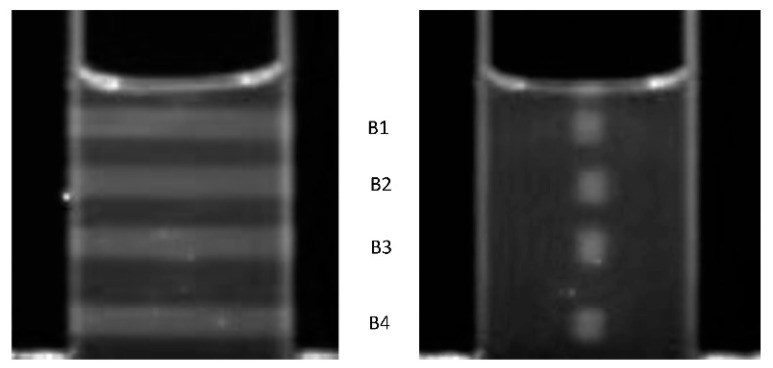
Images taken with Fluorotome 1 of a 20 × 20 mm square, ±30 mm long RFG gel that has been irradiated with four 3 mm square 300 kVp x-ray beams, denoted B1-B4 in the figure. All have total entrance doses of 15.9 ± 0.3 Gy with dose rates from 3.9 to 1.3 Gy/min (top to bottom). The plane of the UV-sheet was at the center of the cell (depth 10 mm) and parallel (left) or orthogonal (right) to the x-ray penetration direction.

To test the overall functioning of the apparatus, we have carried out measurements on an RFG gel sample irradiated using our in-house x-ray source (YXLON model Y.MG325/4.5). The gel was contained in a 20 mm square cell and was 30 mm long. It was irradiated with 4, 3 mm square collimated 300 kVp x-ray beams separated vertically by approximately 7 mm center-to-center. The incident dose rates, calibrated using an ionization chamber, varied from 3.9 to 1.3 Gy/min. The exposures were adjusted to give the same (15.9 ± 0.3 Gy) accumulated dose. Images taken using FluoroTome 1 with the UV sheet at the center of the cell are shown in Figure 15 with the x-ray beam direction parallel or perpendicular to the plane of the UV sheet.

**Figure 16:**
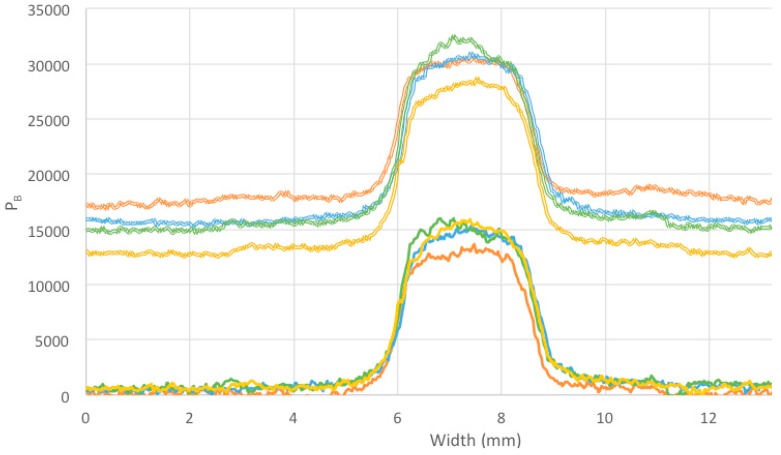
Linear pixel profile scans through the irradiated areas of image 15 (right) without, upper, and with, lower, baseline subtraction. The colour code is orange, B1; blue, B2; yellow, B3; green, B4. The total dose for all beams was 15.9 ± 0.3 Gy with dose rates 3.9, 2.7, 2.0, 1.3 Gy/min for B1, B2, B3, B4.

Linear pixel profile scans, P_B_ versus distance, across the irradiated areas in Figure 15 (right) are shown in Figure 16, with and without background subtraction.

The following parameters derived from the profiles in Figure 16 are listed in Table 2: The maximum increase in intensity (pixel grey scale level, P_B_) on irradiation measured at the center of the beam cross section, ΔI(10) = [P_max_ ‒ P_bkgd_](10); the FWHM of the beam image cross section, W; the 20–80% rise and fall of the beam image (the “penumbra”), δW.

The decrease in ΔI, by 16%, in going from a dose rate of 1.3 to 3.9 Gy/min is much greater than the 4% found between 1.3 and 2.7 Gy/min. We conclude that the dependence on dose rate is not strong but, because of the high background levels and signal noise in the present results, a more thorough investigation of this dependence should be carried out.

The FWHM of the fluorescence resulting from energy deposition by the 3.0 mm square collimated x-ray beams has a half-width of 2.7 mm with a 20–80% rise and fall of 0.49 mm. This 0.49 mm “penumbra” value is a measure of the overall sub-millimeter spatial resolution of detection including beam diffusion. The ultimate resolution possible is expected to be that corresponding to the interpixel distance, which is 0.15 mm/pixel for the present images.

**Figure 17:**
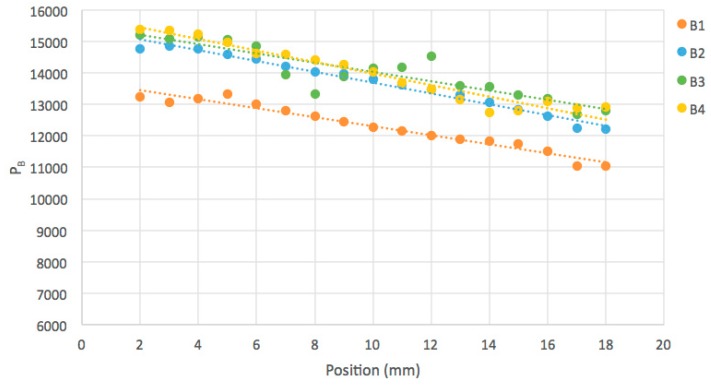
The radiation-induced fluorescence as a function of penetration depth along the x-ray beam for the four beams shown in Figure 15. The ratios of the value at 18 mm to that at 2 mm are listed in Table 2.

In scanning the gel, images were taken every millimeter along the beam axis and the value of the radiation-induced fluorescence, ΔI(z), as a function of penetration depth z is plotted in Figure 17 for the four beams. Values for distances less than 2 mm from the cell walls have been omitted because of optical artifacts close to the interfaces. The ratio of the intensity at 18 mm to that at 2 mm is given in Table 2. This decrease with depth by a factor of 0.836 can be ascribed to attenuation of the x-ray beam over 1.6 cm in the medium i.e.,

ΔI(18)/ΔI(2) = exp[−1.6ρμ](1)

In (1), ρ is the density of the medium, 0.91 g/cm^3^, and μ is the mass attenuation coefficient in g/cm^2^. With ΔI(18)/ΔI(2) = 0.836, μ is 0.123 g/cm^2^ for the 300 kVp x-rays used. Values of 0.154 g/cm^2^ [12] and 0.163 g/cm^2^ [8] for a similar tertiary-butyl acrylate gel have been determined previously for 200 kVp x-rays. The values found are close to those of 0.115, 0.138 and 0.164 g/cm^2^ reported for 300, 200 and 100 keV x-rays for the chemically similar compound polymethylmethacrylate (PMMA) [13].

**Figure 18:**
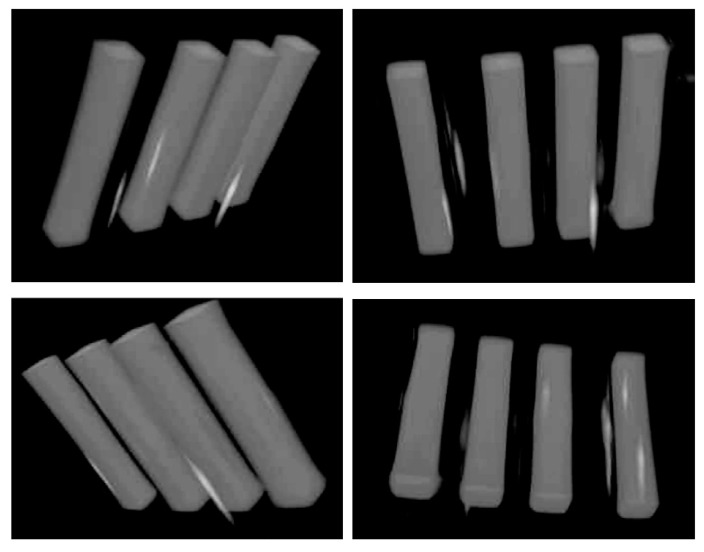
Still frames from the 3D video reconstructed from 20 tomographic images taken of the irradiated gel along the axis of the beams. The bright areas are related to spurious fluorescent dust particles in the gel.

The procedure for forming 3D video images of the fluorescence within the irradiated gel from a series of tomographic images has been described in a previous publications [7,12]. A 3D video of the present irradiated gel, constructed from 20 images taken along the axis of the beams is given in the supporting information. In Figure 18, four still frames from the video are shown. The bright spots that appear outside of the irradiated beams arise from spurious fluorescent dust particles in the gel. They emphasize the importance of physical as well as chemical purity of the gels at all stages of their preparation.

## 6. Conclusions

FluoroTome 1 has been found to be capable of fulfilling the following product specifications:

(1) producing a thin sheet of uniform ultraviolet light.
(2) transporting a liquid or gel medium through this UV sheet using a remote control translation stage over a distance of at least 50 mm.
(3) making multiple digital photographic images of the fluorescence of an irradiated medium produced by the UV excitation as the sheet is scanned through the medium.
(4) having a light-tight encasement; no darkroom requirement.
(5) being readily portable and transportable with no special local facilities required.
(6) capable of preliminary data processing on-site, within minutes of radiation exposure of a gel medium.
(7) having a user-friendly software interface.

It is intended to apply RFG gels and FluoroTome 1 to the study of energy deposition by proton (pencil) beams produced at the Holland Proton Therapy Centre which was recently constructed on a site adjacent to the Reactor Institute of the Delft University of Technology. In addition to fundamental studies of parameters affecting energy deposition, more general applications to dosimetric aspects of quality control and personnel training in radiotherapy are envisaged. Other formulations of radio-fluorogenic gels than that used by the present authors have been reported [14,15]. These should also be amenable to tomographic measurement using FluoroTome 1.

## Figures and Tables

**Table 1:** The UV intensity, measured using the photodiode, of light transmitted by the left and right side slits as a function of height above the cell holder.

Probe Height (mm)	Intensity (mW/cm^2^)		
	Left	Right	Mean
20	10.2	9.6	9.9
30	11.2	10.5	10.9
40	11.0	10.4	10.7
50	11.2	10.9	11.1
60	11.5	11.0	11.3
70	11.1	11.0	11.1
80	11.1	11.0	11.1
90	10.4	11.2	10.8
100	3.0	6.5	4.8

**Table 2:** The parameters derived from the pixel profile scans in Figure 16, the symbols are defined in the text.

Beam	Dose Rate, D’ (Gy/min)	Dose, D (Gy)	ΔI(10)	ΔI(18)/ΔI(2)	W (mm)	δW (mm)
B1	3.9	15.6	12,800	0.834	2.62	0.41
B2	2.7	16.2	14,600	0.827	2.65	0.52
B3	2.0	16.0	15,000	0.841	2.64	0.50
B4	1.3	15.6	15,200	0.841	2.71	0.54
averages		15.9		0.836	2.66	0.49

## Supplementary Materials

Supplementary Materials: The following are available online at https://www.mdpi.com/2073-4360/11/11/1729/s1.
