# Supplementary material for: FluoroTome 1: An Apparatus for Tomographic Imaging of Radio-Fluorogenic (RFG) Gels
Source: Polymers (Basel). 2019 Oct 23;11(11):1729. doi: 10.3390/polym11111729 (PMC6918256; doi:10.3390/polym11111729)
Supplement: Supplementary file 1 [file polymers-11-01729-s001.zip › SP-FINAL/User Manual.pdf]

FluoroTome 1 User Manual

(26/06/2019)

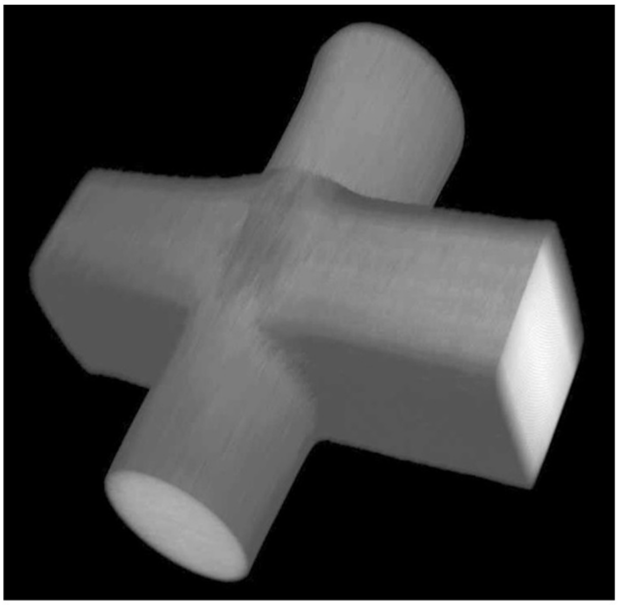

FLUOROTOME

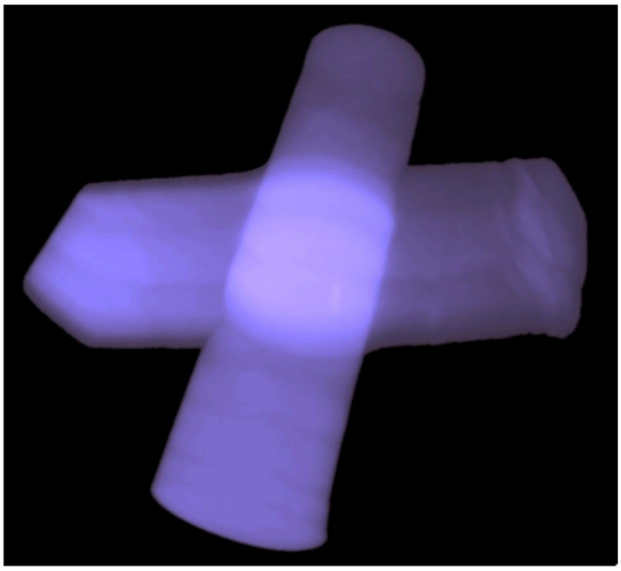

TABLE OF CONTENTS

1. Introduction 3

1.1. The fluorotomography principle 3

1.2. The FluoroTome 1 apparatus 4

2. Getting Started 6

3. Imaging Applications 7

3.1. Actinometry 7

3.2. Single Image 9

3.3. Tomographic Scan 10

4. Bibliography 13

Cover: Still-images from 3D movies of 1 cm crossed square and round x-ray beams in a radiofluorogenic gel using the prototype of FluoroTome 1 [1,2].

1. Introduction

The FluoroTome1 apparatus was constructed by 4PICO BV (Sint Oedenrode, NL) and delivered to the Reactor Institute at the Technical University of Delft on September 10th 2018. The apparatus was designed to be a user-friendly and transportable method of making tomographic images of the fluorescence of radio-fluorogenic (RFG) gels that could be used to perform 3D dosimetry of complex radiation fields. The apparatus is based on the prototype developed and tested in the Applied Radiation and Isotopes (ARI) group of the Reactor Institute Delft [1,2]. In figure 1.1 is shown a photograph of the assembled apparatus including the laptop containing the control and readout software. This version of the manual is based on completely revised software written in January 2019 by Simon Thiele of 4PICO in collaboration with John Warman and Tiantian Yao.

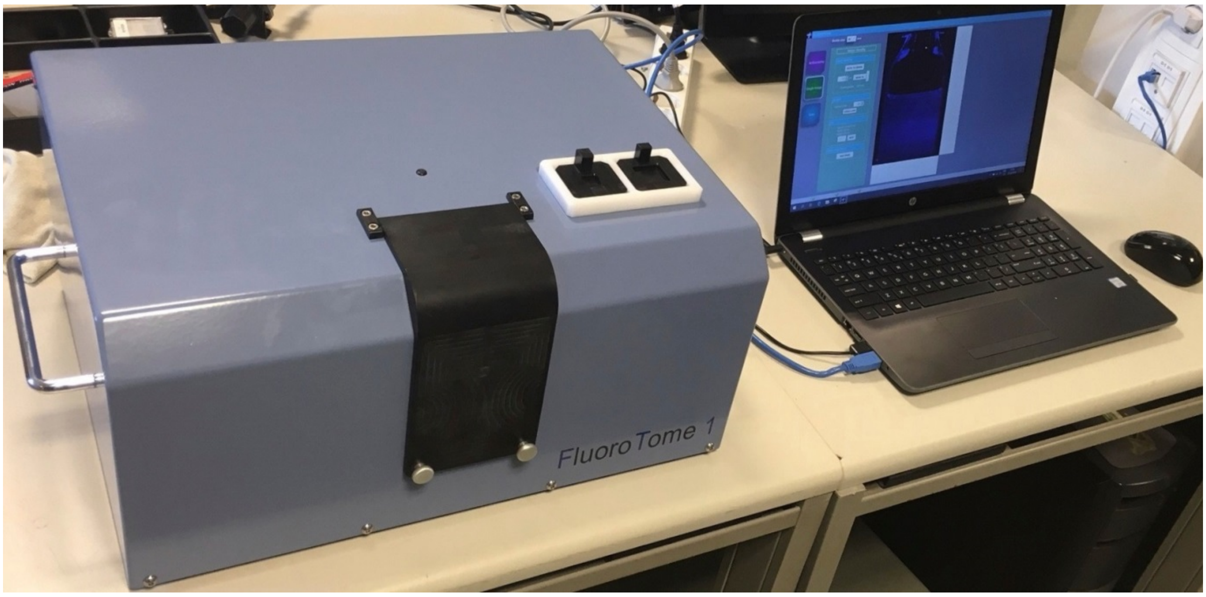

Figure 1.1 Photo of the FluoroTome 1 apparatus, including the laptop containing the control and data storage software. To the right of the opening flap are the adapters for 10 and 20 mm square cells.

1.1. The fluorotomography principle

The operational fluorotomographic principle is illustrated by the schematic drawing of the prototype shown in figure 1.2.

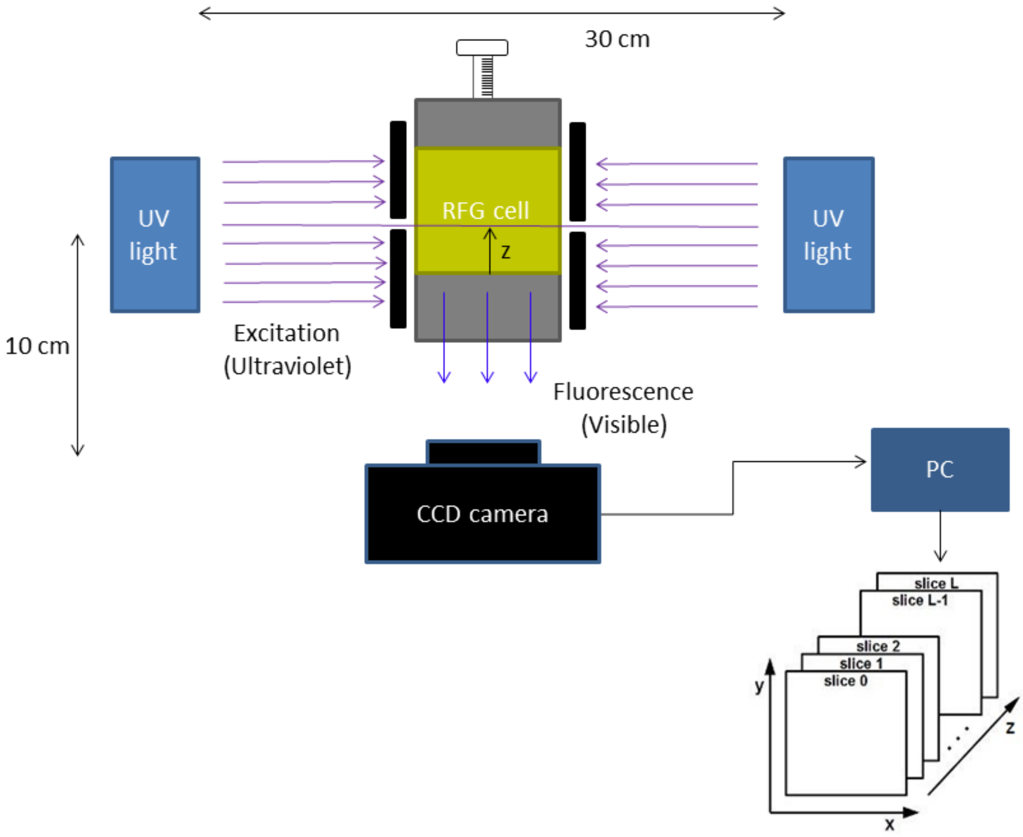

Figure 1.2 Schematic drawing of the main components and mode of operation of the prototype on which FluoroTome 1 is based.

A cell containing a radiofluorogenic gel is placed on a translation stage by which it can be moved past narrow slits in plates on the left and right. The plates are illuminated with collimated beams of UV light from lamps containing vertical arrays of LEDs. In this way a narrow sheet of UV excitation light is created between the plates through which the medium moves. The fluorescence of the sample initiated by the UV sheet is monitored with a digital camera that takes images at given distances of movement. After transference to a computer, the files are compiled and used to construct 3D images of the fluorescence within the sample.

The main application intended for the present apparatus is to produce 3D images in radiofluorogenic (RFG) gels of complex high-energy radiation fields, in particular those produced by modern radiotherapy procedures using high energy photon and particle beams. The results can be used to control computer driven protocols and apparatus functions. A role in the teaching of clinical personnel can also be envisaged. The images are produced within a few minutes of irradiating a gel phantom.

The basic physical chemistry underlying RFG gels has been published together with previous applications to 2D bulk studies of a variety of radiotherapy procedures [3].

1.2. The FluorTome 1 apparatus

In figures 1.3 and 1.4 are shown schematic drawings of the apparatus encased and open respectively.

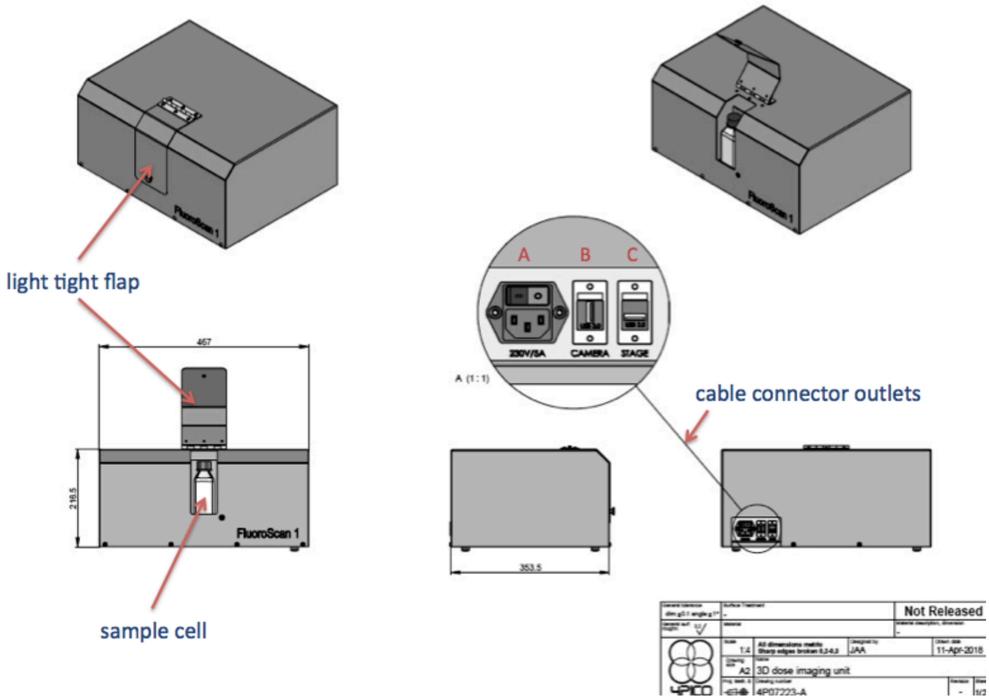

Figure 1.3 The FluorotTome 1 apparatus encased.

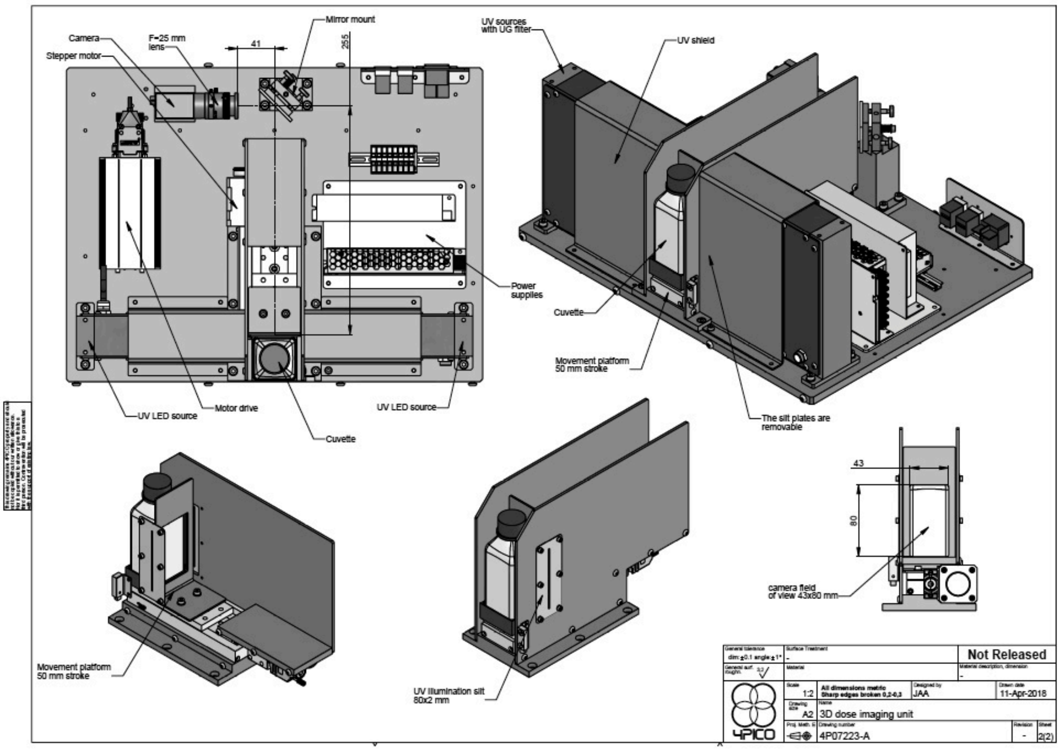

Figure 1.4 The FluoroTome 1 apparatus with encasement removed.

The apparatus is connected to a 220V source via outlet A in figure 1.3 and to the laptop computer for control and read-out via connectors B and C using the cables supplied. RFG gel samples in square glass cells with widths of 40, 20, and 10 mm, can be inserted via the front flap opening into corresponding-size cell holders. Closing the flap results in a completely light-tight interior.

Inside the light-tight box two UV LED lamps generate collimated, ~20 mm wide, vertical beams of UV light centered at 384 nm that are incident on blackened aluminium plates with 2 mm slits placed on either side of cell. The cell holder is on a moveable stage that allows it to be moved past the slits and through the sheet of UV excitation light. Images of the fluorescence from the gel within the cell are made by a CCD camera (HIK VISION), situated in the back left of figure 1.4, via a 45 degree mirror placed at the end of the stage. The raw output recorded by the camera is transferred to the laptop where it is transformed into a 16 bit TIFF and an 8 bit JPEG image. The maximum range of the stage movement is 50 mm and images can be taken as small as every 1 mm movement of the stage.

2. Getting started

Connect the apparatus and the laptop to 220 Volt outlets. Use the cables supplied to interconnect the apparatus to the laptop via connectors **B** and **C**. Switch on the apparatus with the switch on the back of the apparatus just above the 220 Volt input connection **A**.

Turning on the laptop will result in a screen displaying the FluoroTome icon. To start the imaging software click on the FluoroTome icon on the desktop and the start screen, shown in figure 2.1, will appear.

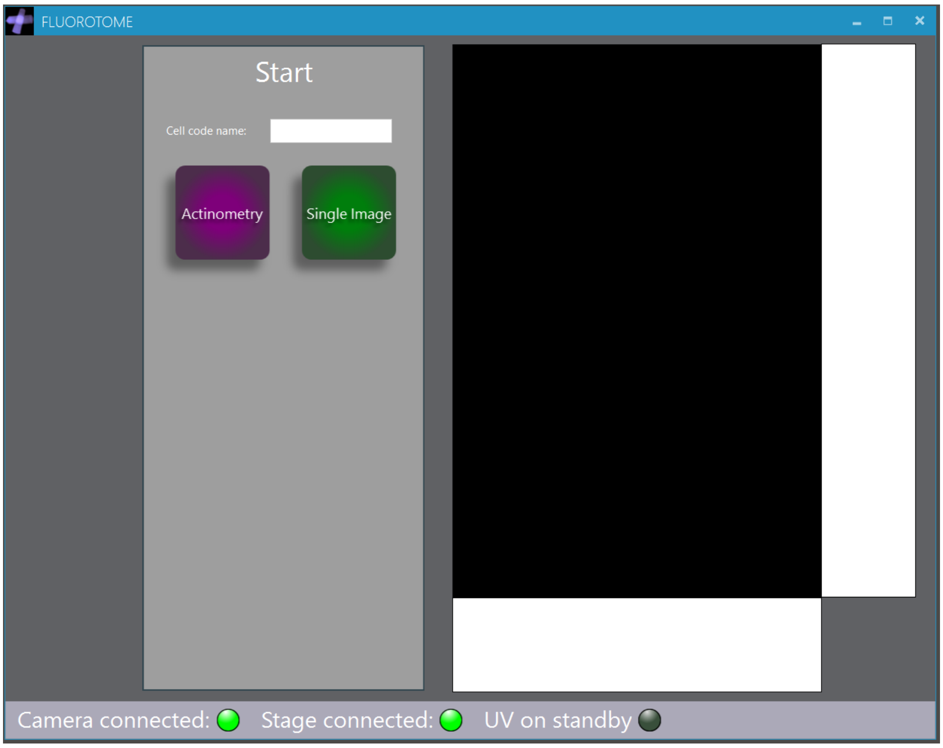

Figure 2.1 FluoroTome start screen

Open the light-tight flap and choose the positioning holder with the dimensions suitable for the particular cell to be measured. The basic, fixed holder is 40x40 mm^2. This can be changed to 20x20 mm^2 or 10x10 mm^2 by inserting the adapters shown in fure 1.1 on the right side of the flap. Place the cell in the holder within the compartment and close the flap. Enter a code name for the cell and then click on either the "Actinometry" or "Single Image" button which starts the imaging software procedures described in the next section.

At the bottom of figure 2.1 are three green traffic lights. The two on the left indicate that the Camera and the Stage are connected. The right hand light is normally out which indicates that the UV lamps are in the default standby mode (i.e. awaiting a computer "ON" command). If the lamps are turned on via a manual override (see section 3.2.) this light comes on as a warning. A safety measure ensures that the lamps cannot be continuously on for more than 5 minutes to prevent overheating.

3. Imaging Applications

Three separate modes of taking images are possible. The first is the "Actinometry" mode that should be routinely used for calibrating the UV light intensity and camera parameters before a series of measurements using RFG gels are made.

3.1. Actinometry

For this a standard dilute solution of Diphenyl Anthracene (DPA) in cyclohexane is used. This should be kept in a cool and dark place. The initial screen displayed in this mode is shown in figure 3.1. The separate elements in the screen menu are discussed below:

STAGE POSITION: This is usually set by default with the UV slits positioned at the center of the of the cell. This should be indicated by "Stage position" 25 mm. If not 25 mm click on "move to center" button. Other positions can be chosen and images stored if so wished.

UV LIGHT: By clicking on the up and down arrows of "exposure time" the effective shutter time of the camera can be changed. A longer exposure time increases the brightness of the image. The maximum exposure time setting is 2.000 seconds. By clicking on "Test Image" the corresponding image is displayed at the right of the menu (figure 3.2.). If a suitably intense image is obtained this can be saved:

STORE ACTINOMETRY: Clicking on "Save Image" stores the image in the actimometry folder. The stored format can be chosen between 8 bit pseudo-JPEG, 16 bit TIFF or both. The file size for typical TIFF image (~30 Mb) is an order of magnitude larger than a JPEG file.

After storing the actinometry image, clicking on the upper left "Back to start" button reverts to the START screen (figure 2.1).

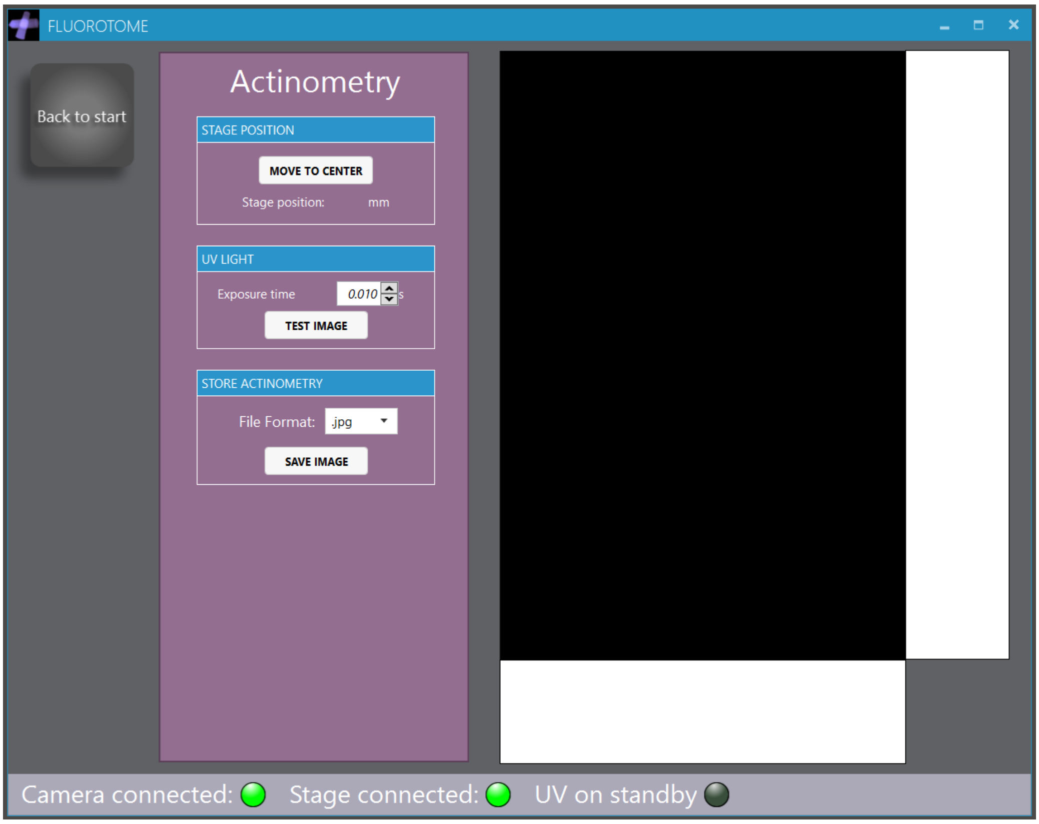

Figure 3.1. Initial Actinometry screen

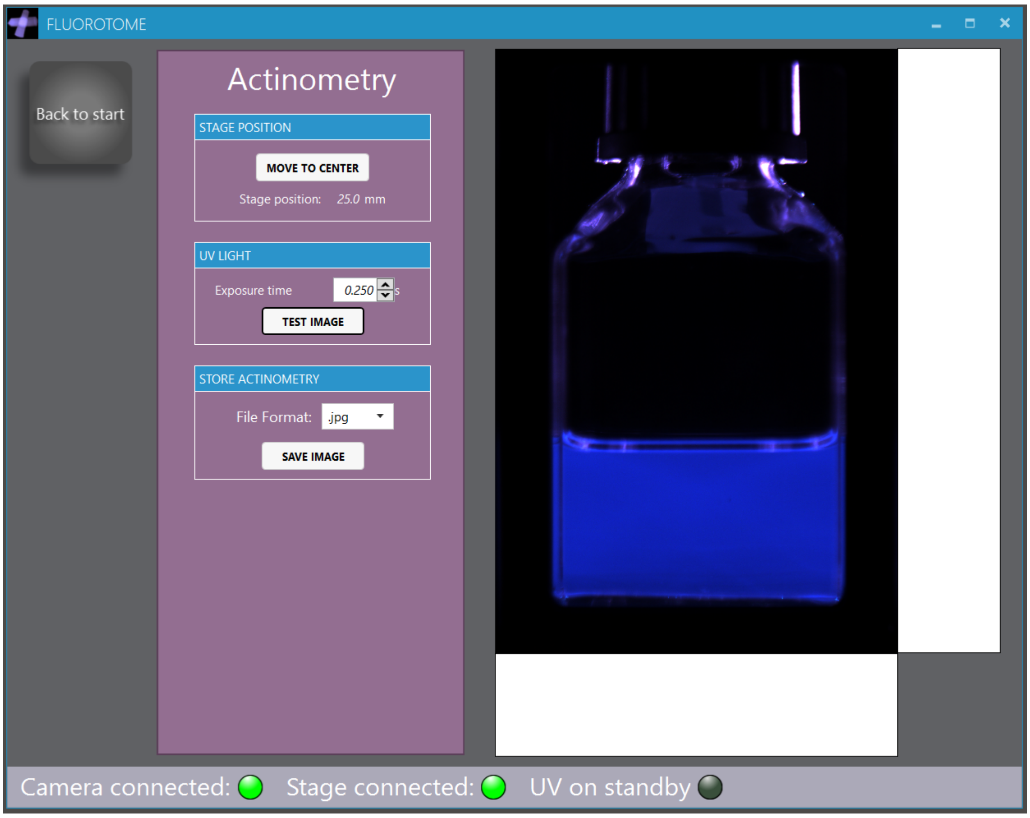

Figure 3.2. A test image for deciding on the optimum exposure time setting.

3.2. Single Image

On returning to the start screen after actinomentry a cell containing a dosimetry medium (prior to or after irradiation) can now be placed in the apparatus holder, its code entered in "cell code" and the button "Single Image" chosen. The single image screen, figure 3.3, is then displayed.

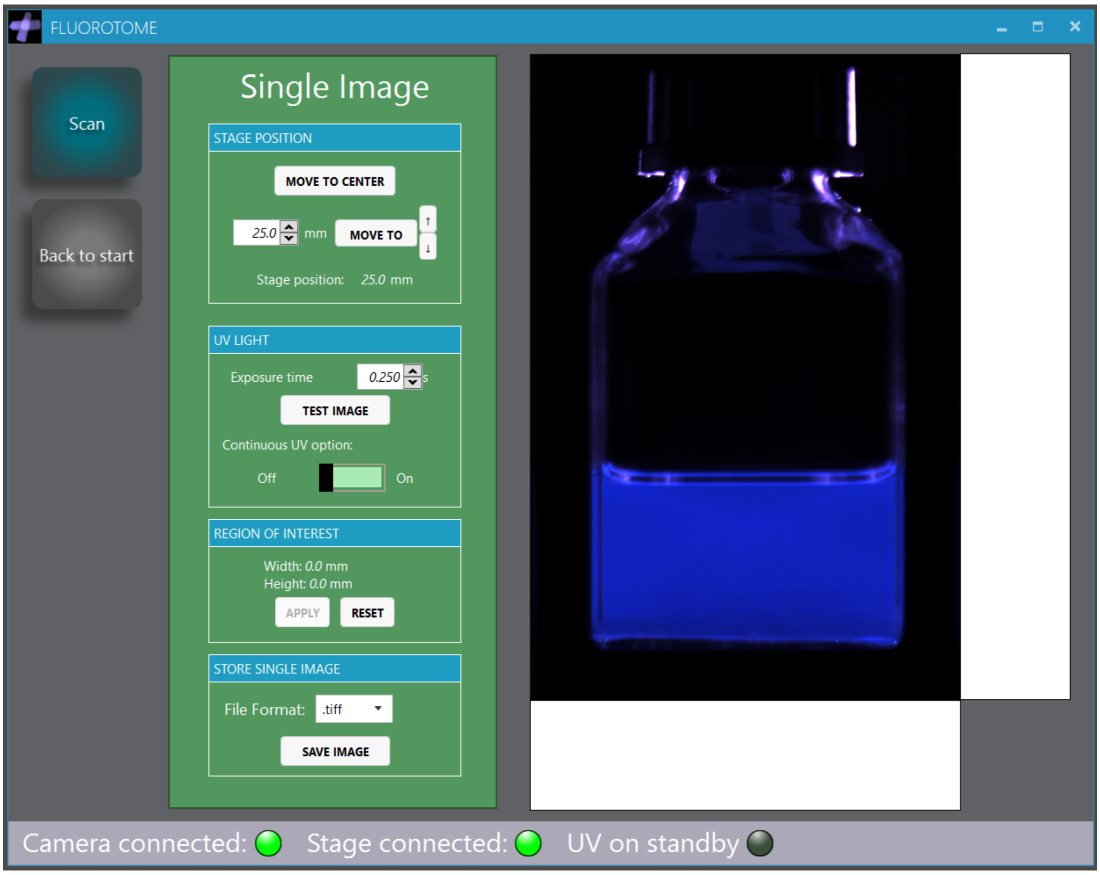

Figure 3.3. The single image screen. On the right is a test image for a given position and exposure time.

STAGE POSITION: The button "move to center" (the default) moves the cell to the position with the UV beam passing through the middle of the cell i.e position 25.0. A specific position can be chosen using "move to". Positions 0.0 and 50.0 are the extreme possible positions away from and towards the operator respectively. By varying the position and observing the "Test Image" in the right-hand window one can decide on the scan limits to be used.

UV LIGHT: An exposure time can be chosen up to 2.000 and a "Test Image" displayed in the window on the right. The exposure time can be changed until a suitable fluorescence intensity and contrast is obtained. This image can then be stored by moving on to "STORE SINGLE IMAGE". It is possible to make images while the UV lamps are on continuously by switching the continuous UV option "ON". In this position the "UV on standby" traffic light will turn on. This is INCORRECT since

when this traffic light is on it signifies that the UV lamps are in fact on continuously. Because of lamp heating the lamps are however always turned off by default after 5 minutes.

REGION OF INTEREST: Choosing this allows one to select a region in the full image that is of interest (and to be saved). An example is shown in figure 3.4.

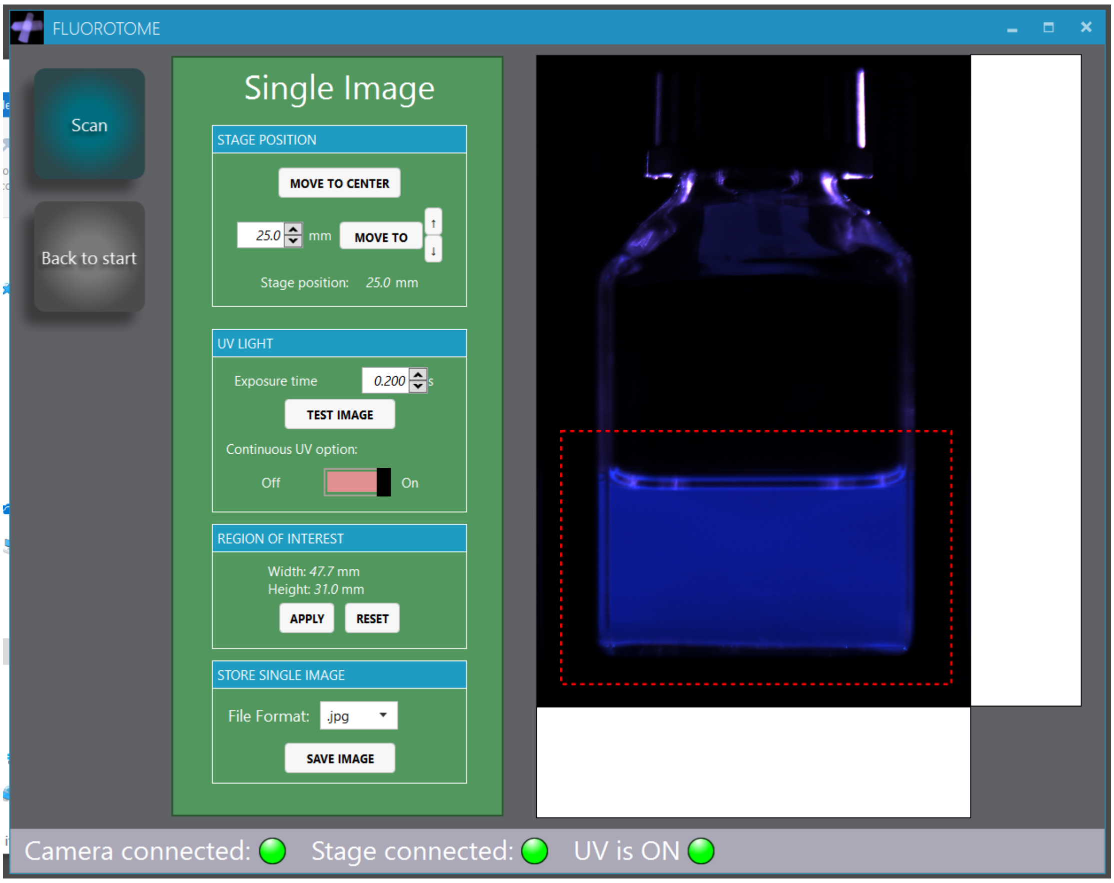

Figure 3.4. The single image screen with a region of interest (ROI) chosen and displayed in the right hand side window. This ROI will be saved. This image happens to be taken with the UV lamps on continuously which is not advised!

STORE SINGLE IMAGE: Before storing the image the method of storage can be chosen; either 8 bit JPEG, 16 bit TIFF or both. Since the JPEG format used appears to be simply 8 bit down on the TIFF (ie not true JPEG) it seems pointless to always store large TIFF files. Both formats show a linear relationship with fluorescence intensity up to saturation. Pressing "save image" will store the image at the latest position, exposure time, and ROI chosen. These parameters will also be used in a subsequent scan of the present cell.

3.3. Tomographic scan

Clicking on the "SCAN" button in the upper left of the single scan screen will open the screen shown in figure 3.5.

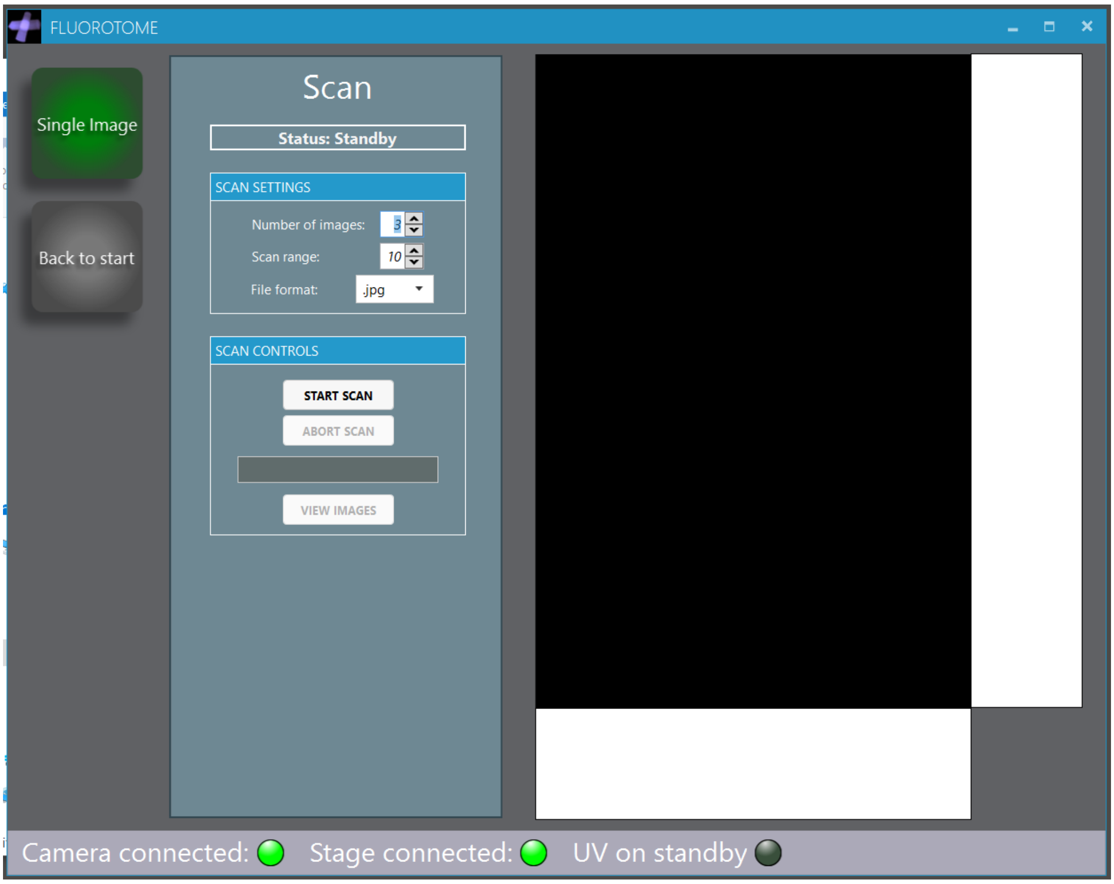

Figure 3.5. The screen for choosing the scan parameters; total range (scan limits) and number of images per scan.

SCAN SETTINGS: The settings to be used will be decided by the geometry of the sample and the radiation field used and possibly tests made in single image mode. The scans will be symmetrical about the central position of the stage (i.e. position 25) and the choice is the total range of the scan. For example a "scan range" of 20 mm corresponds to a scan from 15 mm to 35 mm. The total "number of images" taken during the scan can be chosen, remembering to always add one extra image. For example for a scan of 20 mm with images taken every mm chose "Number of images" = 21. The format of the eventual stored images can also chosen in this section with the default 8 bit JPEG.

SCAN CONTROLS: The scan can be started by clicking on "START SCAN" and aborted by clicking on "ABORT SCAN". The progress of the scanning is illustrated by a moving percentage indicator underneath the abort scan button. The images can be viewed by clicking on "VIEW IMAGES" which opens a screen shown by image viewer in figure 3.6.

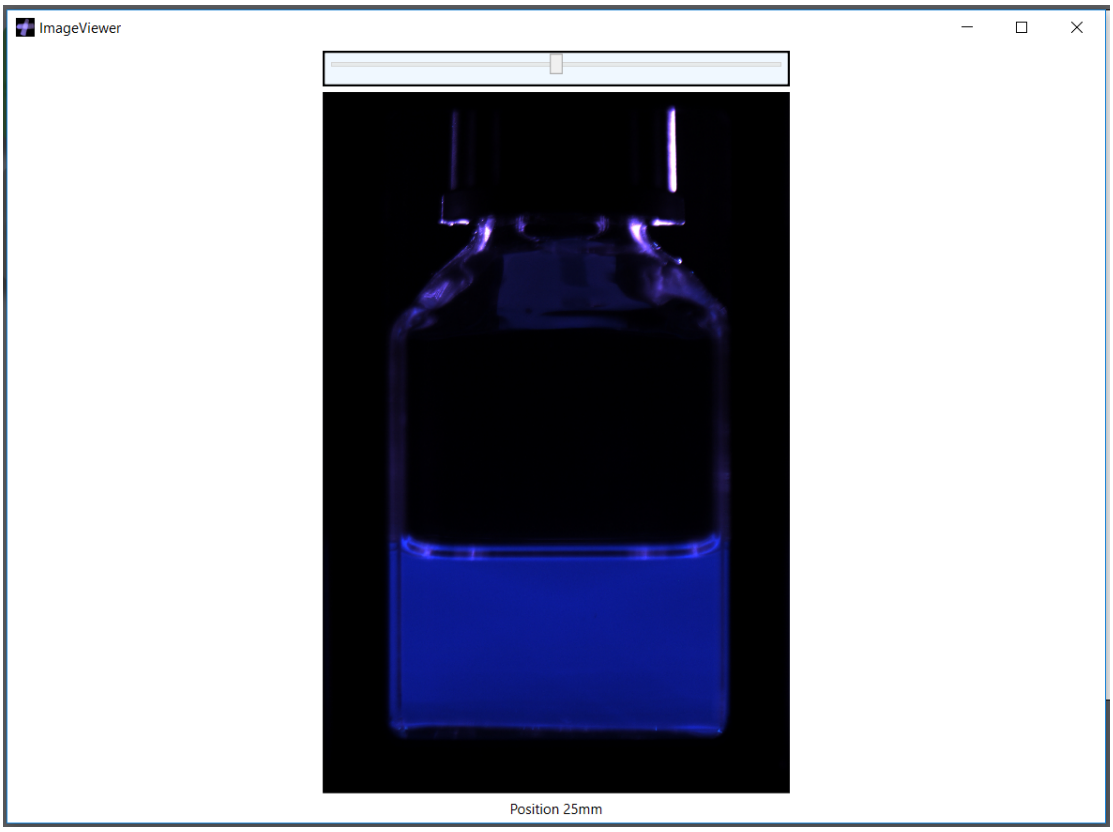

Figure 3.6. Individual images from back to front of the scan can be chosen using the scroll bar above the image with the position chosen given beneath the image.

4. Bibliography

1. Yao, T. 3D radiation dosimetry using a radio-fluorogenic gel.
PhD Thesis, Technische Universiteit Delft, Delft, The Netherlands. 16 January 2017.
https://doi.org/10.4233/uuid:e8590e7e-944c-4b4c-bc2b-3843400a9f85

2. Yao, T.; Gasparini, A.; De Haas, M.P.; Luthjens, L.H.; Denkova, A.G.; Warman, J.M. A tomographic UV-sheet scanning technique for producing 3D fluorescence images of x-ray beams in a radio-fluorogenic gel. *Biomed. Phys. Eng. Express* **2017**, *3*, 027004. https://doi.org/10.1088/2057-1976/aa684b

3. Warman, J.M.; de Haas, M.P.; Luthjens, L.H.; Denkova, A.G.; Yao, T. A radio-fluorogenic polymer-gel makes fixed fluorescent images of complex radiation fields. Polymers, 2018, 10(6), 685. doi:10.3390/polym10060685
