# Supplementary figures and images for: FluoroTome 1: An Apparatus for Tomographic Imaging of Radio-Fluorogenic (RFG) Gels
Source: Polymers (Basel). 2019 Oct 23;11(11):1729. doi: 10.3390/polym11111729 (PMC6918256; doi:10.3390/polym11111729)

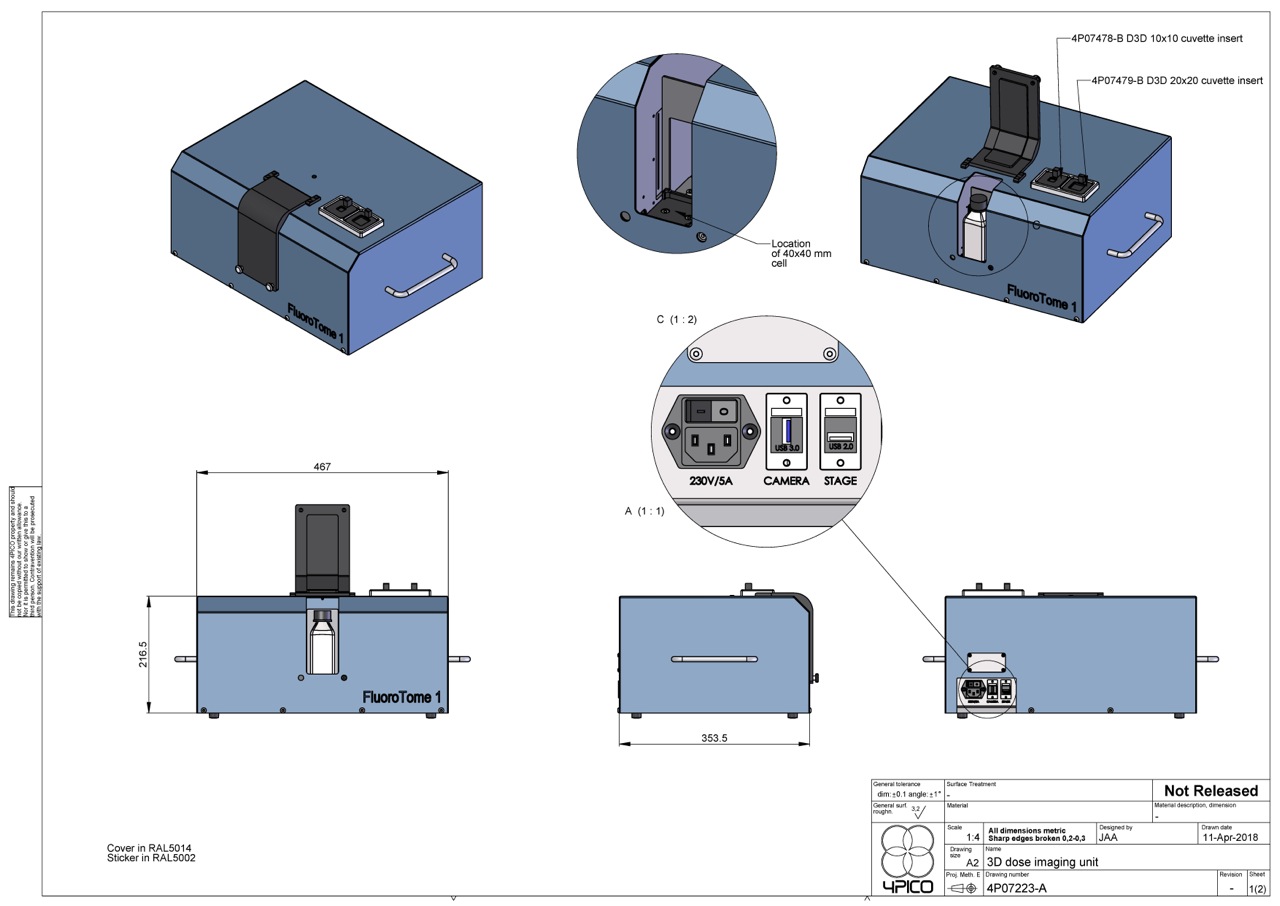

Supplement: Supplementary file 1 [file polymers-11-01729-s001.zip › SP-FINAL/external schematic.jpeg]

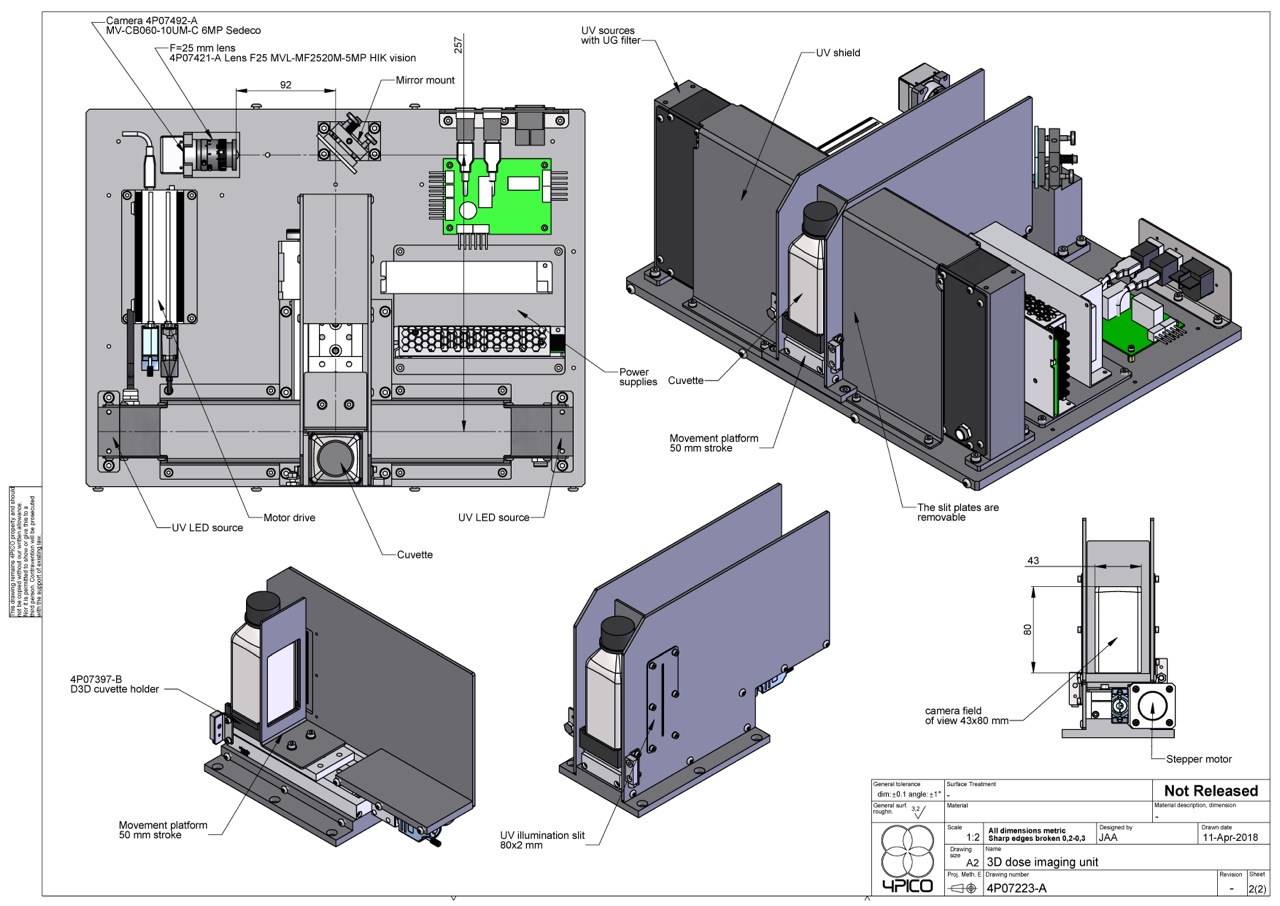

Supplement: Supplementary file 1 [file polymers-11-01729-s001.zip › SP-FINAL/internal schematic.jpeg]

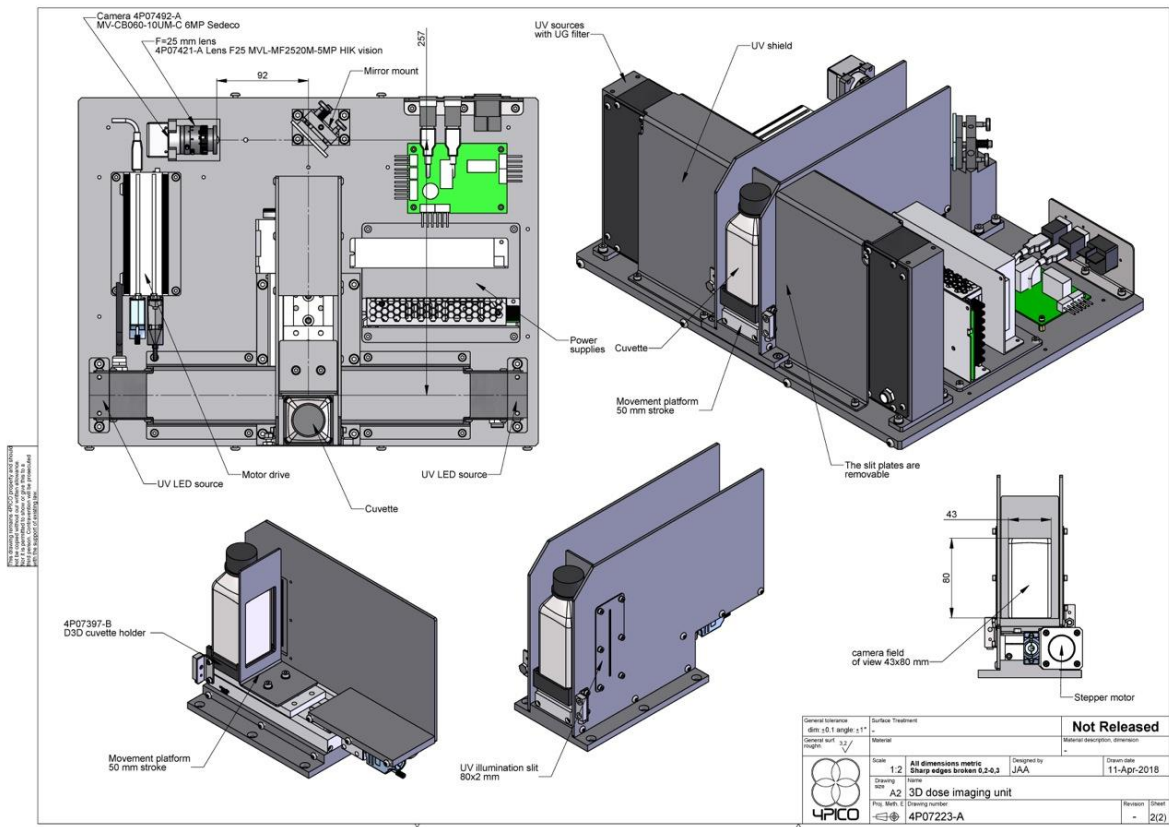

Supplement: Supplementary file 1 [file polymers-11-01729-s001.zip › SP-FINAL/internal schematic.pdf]
