# Supplementary material for: FluoroTome 1: An Apparatus for Tomographic Imaging of Radio-Fluorogenic (RFG) Gels
Source: Polymers (Basel). 2019 Oct 23;11(11):1729. doi: 10.3390/polym11111729 (PMC6918256; doi:10.3390/polym11111729)
Supplement: Supplementary file 1 [file external schematic.pdf › SP-FINAL/external schematic.pdf]

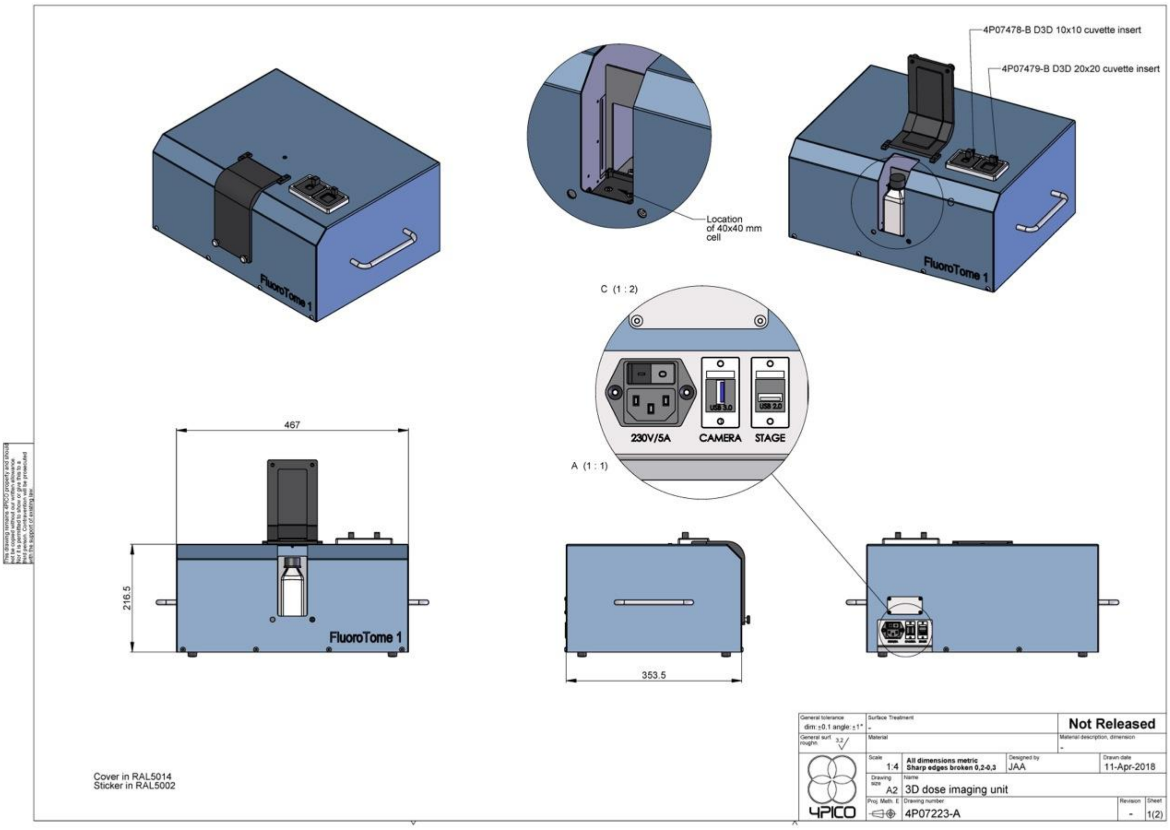
4P07478-B D3D 10x10 cuvette insert
4P07479-B D3D 20x20 cuvette insert
Location of 40x40 mm cell
FluoroTome 1
C (1 : 2)
A (1 : 1)
230V/5A
CAMERA
STAGE
USB 3.0
USB 2.0
467
216.5
353.5
Cover in RAL5014
Sticker in RAL5002
General tolerance
dim: ±0.1 angle: ±1°
Surface Treatment
Not Released
General surf. roughn. 3.2
Material
Material description, dimension
Scale 1:4
All dimensions metric
Sharp edges broken 0,2-0,3
Designed by JAA
Drawn date 11-Apr-2018
Drawing size A2
Name 3D dose imaging unit
Proj. Meth. E
Drawing number 4P07223-A
Revision -
Sheet 1(2)
4PICO
